# *In vitro* metabolic zonation through oxygen gradient on a chip

**DOI:** 10.1038/s41598-019-49412-6

**Published:** 2019-09-19

**Authors:** Federica Tonon, Giovanni Giuseppe Giobbe, Alessandro Zambon, Camilla Luni, Onelia Gagliano, Annarosa Floreani, Gabriele Grassi, Nicola Elvassore

**Affiliations:** 1 Dept. of Life Sciences, University of Trieste, Trieste, 34127 Italy; 2 Stem Cell and Regenerative Medicine Section, UCL GOS Institute of Child Health, London, WC1N 1EH UK; 3 Dept. of Industrial Engineering, University of Padova, Padova, 35131 Italy; 4 Shanghai Institute for Advanced Immunochemical Studies (SIAIS) ShanghaiTech University, Shanghai, 201210 China; 5 Dept. of Surgery, Oncology and Gastroenterology, University of Padova, Padova, 35121 Italy; 6 Venetian Institute of Molecular Medicine, Padova, 35129 Italy

**Keywords:** Embryonic stem cells, Fluidics

## Abstract

Among the multiple metabolic signals involved in the establishment of the hepatic zonation, oxygen could play a key role. Indeed, depending on hepatocyte position in the hepatic lobule, gene expression and metabolism are differently affected by the oxygen gradient present across the lobule. The aim of this study is to understand whether an oxygen gradient, generated *in vitro* in our developed device, is sufficient to instruct a functional metabolic zonation during the differentiation of human embryonic stem cells (hESCs) from endoderm toward terminally differentiated hepatocytes, thus mimicking the *in vivo* situation. For this purpose, a microfluidic device was designed for the generation of a stable oxygen gradient. The oxygen gradient was applied to differentiating hESCs at the pre-hepatoblast stage. The definitive endoderm and hepatic endoderm cells were characterized by the expression of the transcription factor SOX-17 and alpha-fetoprotein (AFP). Immature and mature hepatocytes were characterized by hepatocyte nuclear factor 4-alpha (HNF-4α) and albumin (ALB) expression and also analyzed for cytochrome P450 (CYP3A4) zonation and glycogen accumulation through PAS staining. Metabolic zonated genes expression was assessed through quantitative real time PCR. Application of the oxygen gradient during differentiation induced zonated glycogen storage, which was higher in the hepatocytes grown in high pO_2_ compared to those grown in low pO_2_. The mRNA levels of glutamine synthetase (GLUL), beta-catenin (CTNNB) and its direct target cyclin D1 (CCND1) showed significantly higher expression in the cells grown in low pO_2_ compared to those grown in high pO_2_. On the contrary, carbamoyl-phosphate synthetase 1 (CPS1), ALB, the proliferative marker ki67 (MKI67) and cyclin A (CCNA) resulted to be significantly higher expressed in cells cultured in high pO_2_ compared to those cultured in low pO_2_. These results indicate that the oxygen gradient generated in our device can instruct the establishment of a functional metabolic zonation in differentiating hESCs. The possibility to obtain differentiated hepatocytes *in vitro* may allow in the future to deepen our knowledge about the physiology/pathology of hepatocytes in relation to the oxygen content.

## Introduction

Many human tissues exhibit spatial heterogeneity along the blood flow axis in terms of cell morphology, transcriptomic/proteomic profile and the regulation of metabolic activities in both health and disease. This heterogeneity can be ascribed to the presence of different gradients, such as oxygen, cytokines, nutrients, and specific signaling events during the development, which promote a multifaceted pattern of tissue behaviors^1^.

The liver is probably the most representative organ where spatial heterogeneity takes place. The liver tissue shows changes in functional and metabolic activities across the hepatic sinusoid, a phenomenon known as “zonation”, which occurs from the periportal to the perivenous region. Due to zonation, a different distribution through the liver lobule of nitrogen, carbohydrate, xenobiotic, other metabolites and protein synthesis occurs^1^. Particularly relevant for proper hepatocyte differentiation and specialization/maturation, as well as for the creation of the metabolic zonation, is the presence of an oxygen gradient. *In vivo*, hepatocytes are exposed to an oxygen gradient that influences gene expression, metabolism, and produces a considerable heterogeneity and functional plasticity in liver parenchyma^2^. Cells in the periportal zone of the hepatic lobule, receiving oxygen-rich blood (O_2_ partial pressure 60–70 mmHg) from the hepatic artery and nutrient-rich blood from portal vein, catalyze predominantly oxidative energy metabolism using fatty acid β-oxidation and amino acid catabolism^1,3^. In contrast, the perivenous hepatocytes, being in the proximity to the central vein, receive poorly oxygenated blood (O_2_ partial pressure 25–35 mmHg) and activate mainly the metabolic pathways of glycolysis and liposynthesis^1,3^. Somehow peculiar is the metabolic pathway leading to the synthesis of glycogen. While in the perivenous zone, glycogen synthesis is activated when the starting substrate is glucose, in the periportal zone glycogen synthesis is promoted when the starting substrate is lactate^2^. In addition, the synthesis of certain plasma proteins, such as albumin, occurs mainly in the periportal area^4^. Recently, it has been suggested the master role of the Wnt/beta-catenin pathway in the direct regulation of the gene expression pattern on which the metabolic zonation is probably based^1,5^. For example, Wnt/β-catenin pathway seems to regulate the expression of many genes involved in the urea cycle and glutamine synthesis, including CPS1 and GLUL. In particular, these two transcripts are upregulated and downregulated in the periportal and perivenous region, respectively^4–6^. Moreover, the proliferation rate of the periportal hepatocytes results to be higher compared to that of the perivenous hepatocytes^7^.

The availability of *in vitro* cultured hepatocytes stably resembling the heterogeneity of phenotypes of liver hepatocytes *in vivo*, could be of great advantage to study the physiological and the pathological behaviors of the different population of hepatocytes. Given the driving role of oxygen gradient in the formation of the hepatic metabolic zonation^8^, the generation of devices able to reproduce the proper oxygen gradient is a prerequisite to mimic *in vitro* the *in vivo* condition. Moreover, because some events of liver zonation start already at the embryonic stage^5^, the use of differentiating hepatocytes in *in vitro* models may provide more physiological information than the use of differentiated hepatocytes.

In the last years, many tools and technologies have been proposed to create spatio-temporal gradients for cell studies^9–12^. Among these, microfluidic is certainly one of the most promising techniques due to its great controllability in both spatial and temporal domains^8,9^. Some devices were developed to establish an oxygen gradient in order to stimulate mature hepatocytes to get a zonation-like phenotype. Other systems allowed the generation of oxygen gradients on cultured primary rat hepatocytes and fibroblasts. In all these cases, cells were exposed to chemical gradient established by continuous perfusion, which, unfortunately, did not allow decoupling the control of medium delivery from the oxygen concentration. Moreover, these studies provided experimental evidences for primary animal cells, but not human hepatocytes^13–15^. In other approaches^1,16^, primary human hepatocytes were applied in oxygen-dependent modelling. However, a model of hepatic zonation and functional maturation starting from human pluripotent cells undergoing hepatic differentiation was never reported before.

In this work we studied the effects of an oxygen gradient, applied during differentiation from endoderm-committed cells to hepatocytes, on the establishment of a functional hepatic zonation. To this purpose, we developed a microfluidic cell culture device capable to generate a stable oxygen gradient, mimicking the one to which differentiated hepatocytes are physiologically exposed, independently from medium perfusion.

Our device can generate an oxygen gradient with long-term stability and full compatibility with biological incubator. The entire microfluidic device is made of glass and polydimethylsiloxane (PDMS), an elastomeric material that is biocompatible, optically clear and inert, and has great gas permeability^17^. O_2_ spatial concentration was probed by fluorescence of a ruthenium-based compound solution to characterize the oxygen gradient formed inside the chambers of the microfluidic device.

As cell model, we employed differentiating human embryonic stem cells. In addition to the oxygen gradient, hESCs were exposed to the conditions we previously showed to induce hepatic differentiation in a microfluidic environment^18,19^. Thus, we had the possibility to study the effects of the oxygen gradient on differentiating hepatocytes. Our data indicate that the microfluidic cell culture device is suitable to generate a stable oxygen gradient. Moreover, the gradient positively affects the phenotype and gene expression of differentiating hepatocytes as it occurs in the different zones of the hepatic lobule.

## Results

### Description and testing of the microfluidic device

**Figure 1:**
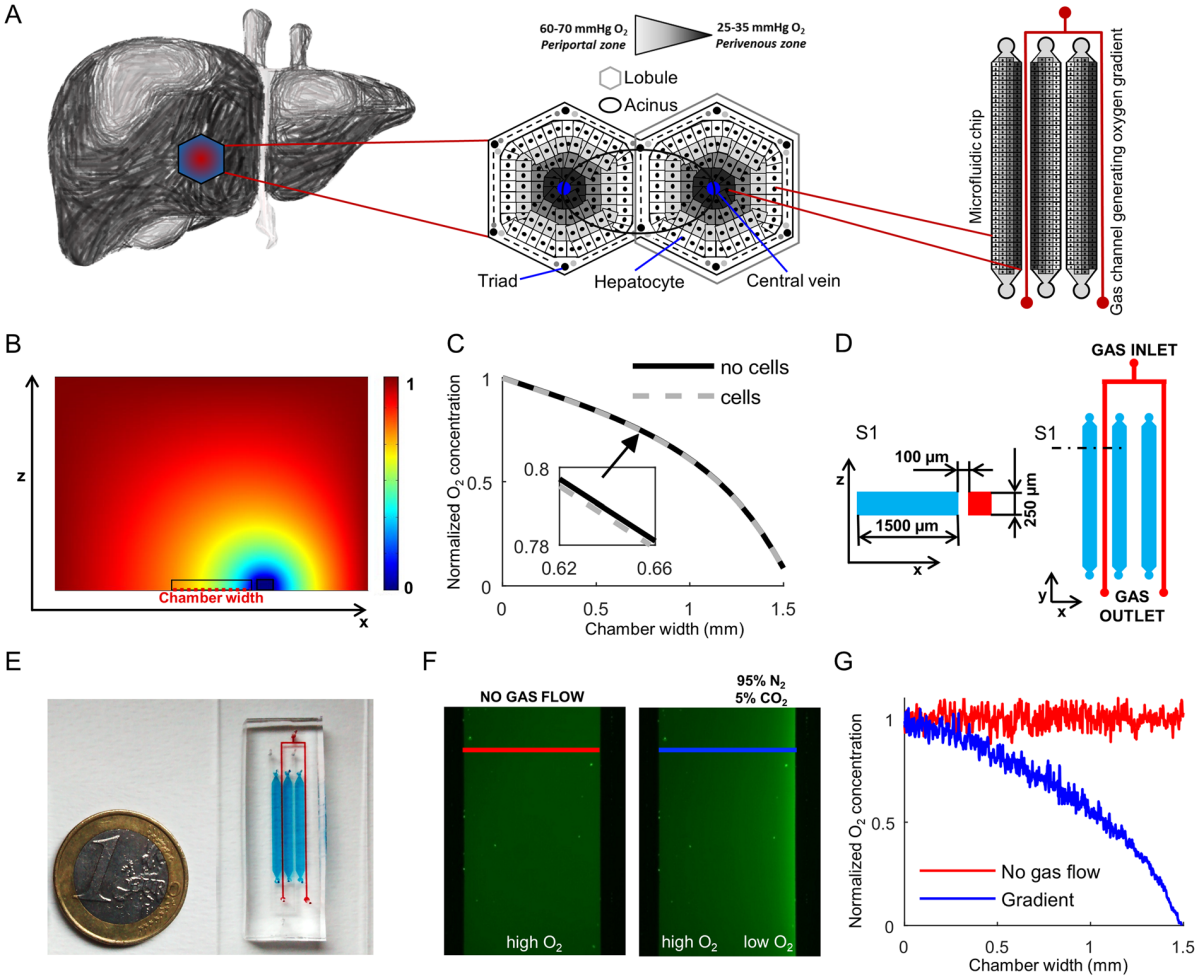
Microfluidic chip design and oxygen gradient validation. (**A**) Representative image of the liver zonation generated across the hepatic lobules. Hepatocytes are naturally exposed to an oxygen gradient according to their position along the portal-central axis that allows distinguishing “periportal” from “perivenous” hepatocytes. Schematic representation of miniaturization process of the sinusoid geometry transferred to microfluidic cultures. (**B**) Simulation results of the steady-state oxygen concentration in the vertical cross section of the PDMS chip. Color bar shows the normalized oxygen concentration (c’) between 0 and 1. The section of cell culture and gas flow channels is also indicated. (**C**) Simulated steady-state profile of the scaled oxygen concentration at the bottom of the culture chamber as indicated in B, with or without cell. (**D**) Schematic section S1 (left) and top view (right) of the microfluidic gradient chip. Red: 95% N_2_/5% CO_2_ gas flow channels. Cyan: culture chambers. (**E**) Picture of the microfluidic chip filled with dyes according to the colors in **D**. (**F**) Fluorescent images of the culture chambers filled with an oxygen-sensitive ruthenium solution in PBS 1X, without gas inflowing into the gas channel (left in red) and at steady-state with nitrogen and carbon dioxide gas mixture flowing at 1 mL/min (right in blue). (**G**) Normalized fluorescent intensity along the culture channel cross-sections highlighted by red and blue lines in F. Red: control condition without gas flow. Blue: steady-state condition with gas flowing at 1 mL/min flow rate at the right side of the microchannel.

To mimic the physiologic condition existing in the liver lobule, we developed a microfluidic cell culture device where hepatocytes are differentiated from hESCs under an O_2_ gradient (Fig. 1A). The microfluidic device includes channel-shaped cell culture chambers whose geometry was previously used for hepatic differentiation from human pluripotent cells^18^. Along one side of the culture chamber, a 95% N_2_/5% CO_2_ gas mixture is continuously flowed; this region is separated from the culture channel by a thin gas-permeable membrane made of PDMS able to produce a localized decrease of oxygen concentration with respect to the incubator gas composition.

We first performed a computer-assisted design of the system to check the spatial control of the oxygen concentration. The computational model was used to simulate the feasibility of producing a stable oxygen gradient across the culture chamber along the whole length of it, and to determine the minimum suitable gas flow rate (Fig. 1B). Simulation results have been computed with an environmental gas composition of 21% oxygen, as in a standard normoxic biological incubator. Oxygen concentration goes from a high level to a low level correspondently to the concentration into the gas channels. The simulated oxygen gradient ranges from 160 mmHg (21%) to 15 mmHg (2%). Cell oxygen consumption was also taken into account in the simulation (Fig. 1C). Figure 1D,E show the setup design and an image of the final chip made of transparent PDMS, attached onto a glass slide. The chip includes three independent parallel culture chambers. Figure 1F shows the experimental validation of the system at steady state. A ruthenium solution was used in the channel as fluorescence sensor of oxygen concentration. The time required for achieving a stable oxygen gradient after starting the gas inflow is less than 1 minute (Suppl. Vid. 1). Ruthenium emits higher green fluorescence at decreasing oxygen concentration. The oxygen profile shown in Fig. 1G was derived with Stern-Volmer equation and indicates that it is possible to obtain different zones in the culture chamber of approximately 1500 µm width: a low oxygen concentration close to the gas channel, an intermediate zone in the middle and a normal oxygen concentration at the opposite side of the channel. The O_2_ profile obtained experimentally is consistent with the simulation of the computational model.

### HESCs can be differentiated into microfluidic channels to obtain hepatocyte-like cells

**Figure 2:**
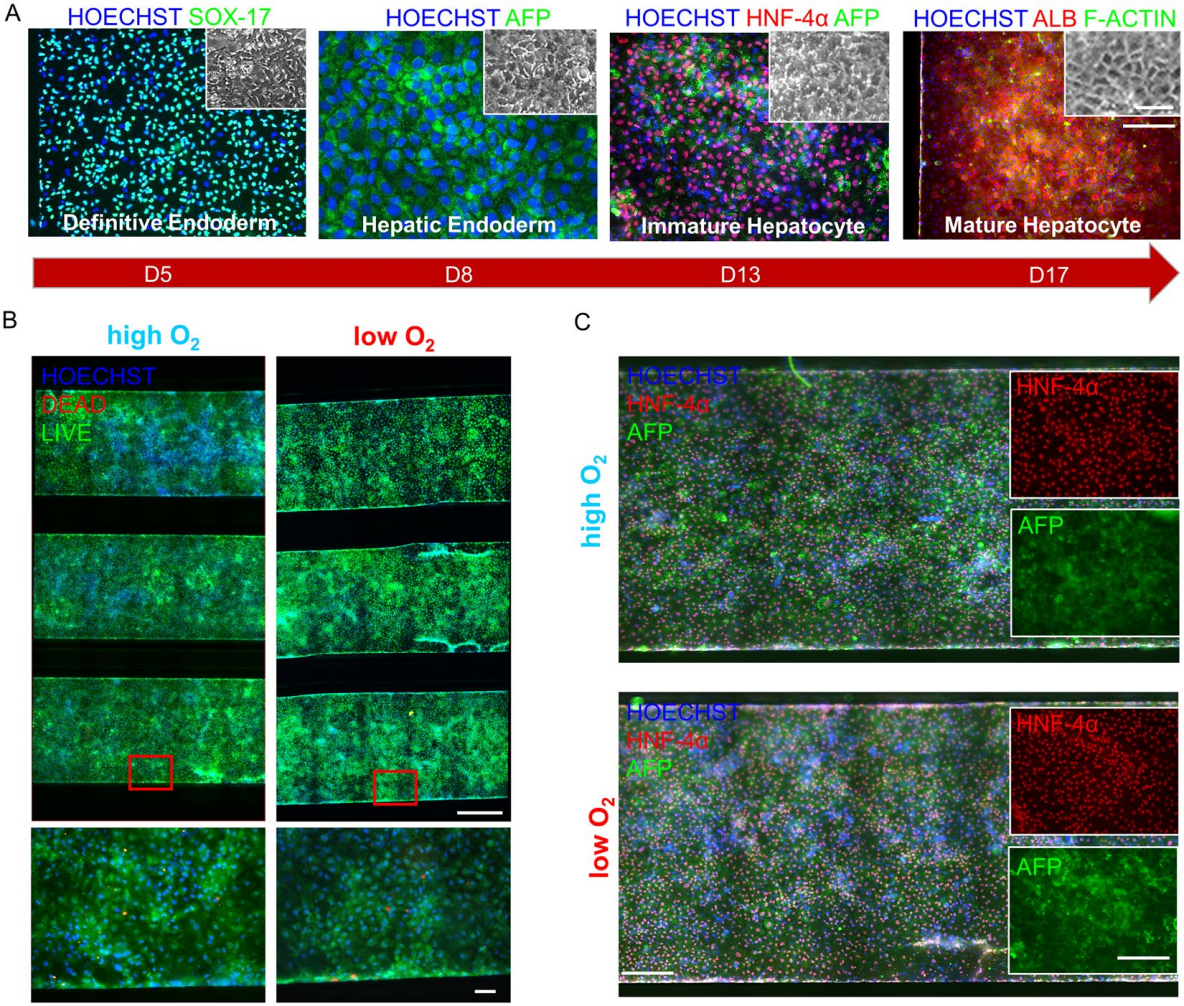
Characterization of hESCs (MShef-3 line) hepatic differentiation into a microfluidic chip. (**A**) Cells showing SOX-17 (green) at day 5, alpha-fetoprotein (green) at day 8, HNF-4α (red) and alpha-fetoprotein (green) at day 13, albumin (red) and f-actin (green) at day 17 of differentiation. Scale bars 200 μm (microchannel) and 20 μm (inset). (**B**) Live/Dead assay showing high (left) and low (right) O_2_ differentiated hESCs at day 12 in microfluidic chips (3 parallel channels above, with enlargement below). Cell nuclei counterstained with Hoechst in blue, dead cells stained in red with ethidium homodimer-1, living cells fluorescence in green with calcein AM. Scale bars 300 μm (microchannels, above) and 10 μm (enlargement, below). (**C**) Day 13 differentiated hESCs showing HNF-4α (red) and alpha-fetoprotein (green), in high (above) and low (below) O_2_ differentiation experiments. Scale bars 100 μm (microchannel) and 50 μm (inset).

By means of the microfluidic device developed, hESCs were differentiated into hepatocyte-like cells under a stable oxygen gradient using a 17-day differentiation protocol we previously described^18^. Cells were characterized for the expression of hepatic differentiation markers, at each stage of the microfluidic differentiation. The cell committed to definitive endoderm (DE) showed SOX-17 homogeneous expression (day 5 of differentiation). Hepatic endodermal (HE) cells showed high expression of alpha-fetoprotein (day 8). Immature hepatocytes (IH) showed homogenous expression of HNF-4α and alpha-fetoprotein (day 13). At day 17 of differentiation, mature hepatocyte-like cells (MH) showed polygonal cell shape, and high and homogeneous expression of ALB (Fig. 2A).

In order to understand whether different oxygen concentration could impair survival or differentiation capacity, we firstly performed separated high (21%) and low (5%) oxygen differentiation experiments. Cells analyzed at day 12 (IH) showed high viability and almost absent cell death in both high and low O_2_ concentrations (Fig. 2B). Furthermore, cells analyzed at day 13 (IH) showed high and homogeneous expression of HNF-4α and alpha-fetoprotein along the channels, in high as well as low O_2_ concentrations (Fig. 2C).

### The stable oxygen gradient in the microfluidic chambers induces metabolic zonation in hESCs-derived hepatocyte-like cells

In order to obtain a measurable effect on the differentiating cells, we applied an oxygen gradient, across the microchannels of the microfluidic device. We observed that the application of the gradient from day 1 of differentiation causes impairment in early differentiation with differential alpha-fetoprotein expression across the channel at day 8 (Suppl. Fig. 1). To avoid any bias in late differentiation, we homogenously differentiated the cells without any gradient, at standard O_2_ (21%) up to the stage of hepatic endoderm (day 8), and then applied the O_2_ gradient until the mature hepatocyte stage (day 17), for a total of 9 days.

**Figure 3:**
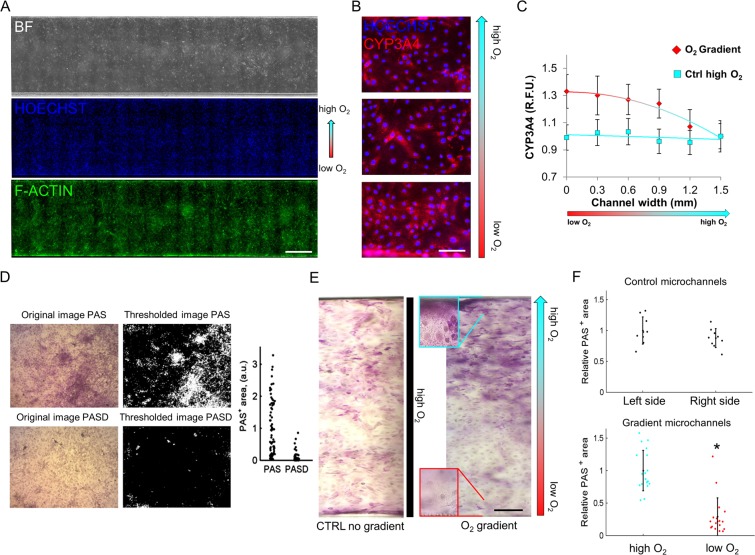
Liver zonation and hepatic glycogen storage evaluation. (**A**) Microfluidic differentiated (HES2 line) hepatocyte under constant oxygen gradient at day 17 of differentiation. Nuclear counterstain with Hoechst (blue) and f-actin staining (green). Scale bar 300 µm. (**B**) CYP3A4 (red) staining with representative images of day 17 differentiated hepatocytes across the gradient microchannel. Scale bar 20 µm. (**C**) Relative fluorescence unit plot of ROI in gradient microchannels and control microchannels (high O_2_ differentiation). Data are expressed as mean ± SD (n > 10) and trend line. (**D**) Example of automated recognition of Periodic Acid-Shiff (PAS+) areas in images taken after PAS staining of hepatocyte-like cells, without or with diastase (indicated with **D**) treatment. (**E**) Representative color images of PAS staining in hepatocyte-like cells grown in control and O_2_ gradient chip (with enlargement inset), respectively. Scale bar 100 µm. (**F**) Quantification of PAS+ areas in images obtained from the control and O_2_ gradient chip, shown in E. Data were acquired from the central part of the channel to the borders. Data are expressed as mean ± SD (n > 9). *Student’s *t*-test p < 0.05.

We first proved that on the last differentiation day (day 17), mature hepatocytes were homogenously distributed in the microfluidic channels (Fig. 3A). By immunofluorescence analysis we studied the differential expression of the drug-metabolizing enzyme CYP3A4 across the microchannel width (Fig. 3B). CYP3A4 fluorescence was quantified in Regions Of Interest (ROI) localized in the upper (high O_2_), middle (intermediate O_2_), and lower (low O_2_) sections of the gradient channel width; in parallel a quantification of non-gradient channels differentiated at ambient oxygen (high O_2_) was performed. Relative fluorescence unit plot shows a higher CYP3A4 expression in lower oxygen concentration, with a constant decrease in expression as O_2_ increases in the gradient^1,20^ (Fig. 3C).

The physiologic and metabolic effects of the O_2_ gradient on differentiated hepatocyte-like cells were also analyzed by evaluating one of the main markers of zonation *i*.*e*. glycogen synthesis. Glycogen accumulation was quantified implementing an image analysis algorithm that classifies PAS positive or negative pixels according to the image saturation intensity. This algorithm was first trained by comparing pictures of PAS staining obtained from cells treated/untreated with diastase (Fig. 3D). Subsequently, the test was applied to differentiated hepatocyte like cells (Fig. 3E). In this case, higher glycogen storage at the side of the chambers in high pO_2_ compared to low pO_2_ was observed. In contrast, no difference in glycogen storage was observed at the two sides of the control chip channels (not exposed to O_2_ gradient throughout differentiation, Fig. 3F). These findings suggest that in our gradient chips, glycogen synthesis mainly originates from lactate, as it occurs for hepatocytes exposed to high pO_2_ in the periportal zone of the hepatic lobule^2^.

**Figure 4:**
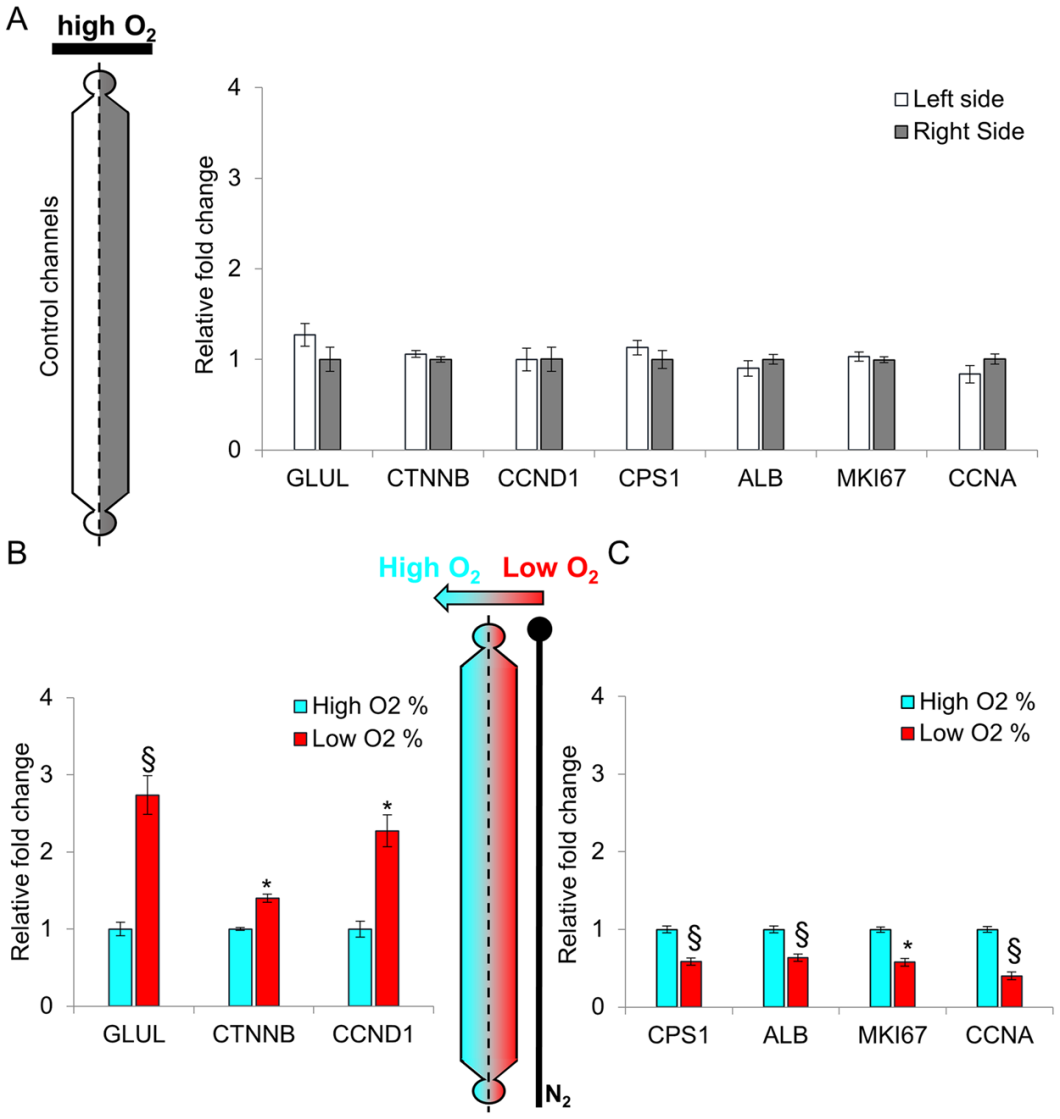
Gene expression analysis in hepatocyte-like cells (HES2 line). In all panels, the levels of the target mRNA are normalized to the expression level of the 28S rRNA. (**A**) Real-time PCR of genes involved in metabolic and cell cycle processes in hepatocyte-like cells obtained following hESCs differentiation at uniform 21% O_2_ concentration (control chip). No statistical difference is observed between the right side and the left side. Data, normalized to the average of the right side, are shown as mean ± SEM. n = 3. Real-time PCR of genes involved in metabolic and cell cycle processes in hepatocyte-like cells grown in a stable O_2_ gradient generated by the microfluidic device. (**B**) GLUL (glutamine synthetase), CTNNB (beta-catenin) and CCND1 (cyclin D1) show significantly higher expression in low pO_2_. (**C**) CPS-1 (carbamoyl-phosphate synthetase 1), ALB (albumin), MKI67, CCNA (cyclin A) show significantly higher expression in high pO_2_. Data, normalized to the average of 21% O_2_, are shown as mean ± SEM. n = 9. p* < 0.001 and p§ < 0.0001 respect to 21% O_2_.

To further investigate whether the induction of a functional differentiated metabolic zonation in hepatic cells is effectively determined by an oxygen gradient, we analyzed the mRNA levels of two genes involved in the urea cycle, GLUL and CPS1, encoding for glutamine synthetase and carbamoyl-phosphate synthetase 1, respectively. Moreover, we evaluated the expression of beta-catenin and its substrate cyclin D1. Finally, we checked the level of proliferation by quantifying cell number and the markers cyclin A and MKI67. Microchannel monolayers of cells were carefully isolated longitudinally, and manually divided into two sections (high and low O_2_) for differential RNA analysis (Suppl. Vid. 2 and Suppl. Fig. 2). No significant changes were observed in hepatocyte-like cells differentiated at constant high pO_2_, between the two sides of the channel (control chip) (Fig. 4A). On the contrary, in the gradient chips, the mRNA levels of GLUL, beta-catenin (CTNNB) and its direct target cyclin D1 (CCND1) showed significantly higher expression in the cells grown in low pO_2_ compared to those grown in high pO_2_ (Fig. 4B). Moreover, CPS1, albumin (ALB), MKI67 and cyclin A (CCNA) resulted to display significantly higher expression in cells cultured at high pO_2_ compared to those cultured in low pO_2_ (Fig. 4C).

Together, these observations indicate that the oxygen gradient generated by our device has a direct effect on the regulation of gene expression. Moreover, the observed regulation is consistent with the oxygen-dependent gene expression found in the different zones of the hepatic lobule^4,21,22^.

The increased expressions of the proliferation markers MKI67 and cyclin A suggest an augmented growth rate of cells in high pO_2_. The increased expression of the G1-phase cycling D1, but not of the S-phase cyclin A in cells maintained in low pO_2_, suggest the incapacity of these cells to progress from the G1 to the S-phase of the cell cycle. To confirm the increased proliferation rate in high pO_2_, we performed cell counting on KI-67 immunostained cells, which revealed a higher number of KI-67 positive cells in high pO_2_ compared to low pO_2_ (Suppl. Fig. 3A). This observation was also confirmed by cell nuclei counting which revealed 16% more nuclei in high pO_2_ compared to low pO_2_ (Suppl. Fig. 3B).

## Discussion

In standard differentiation cultures^23–25^ it is still problematic to accurately reproduce the phenomenon occurring *in vivo* with regard to the differentiation of stem cells towards hepatocyte-like cells. Moreover, hepatic differentiation from human pluripotent stem cells requires long-term experiments and up to 3 weeks could be needed for functional differentiation. Therefore, simple and easy to operate devices are desired from the biological community. The use of advanced technologies can minimize the above problems and better mimicking the *in vivo* differentiation process. Thus, advanced technologies have the potential to allow the generation of hepatocytes usable for drug screening, toxicological assays and may be also as liver disease *in vitro* models.

The O_2_ gradient has a fundamental impact on hepatocytes phenotype and functions across the liver lobule^4–6,26^. Thus, in this work a microfluidic cell culture chip was designed and developed to create a stable oxygen gradient for several days that could be easily operated by simple manual maneuvers or coupled to automatic cell culture liquid handling.

The *in vivo* O_2_ gradient to which sinusoidal hepatocytes are exposed is 60–70 mmHg (8–9%) to 25–35 mmHg (3–4%)^26^. This range is too small for a first demonstration of gradient differentiation, thus, to give the proof of concept and try to observe actual results on our cultures, we enlarged the O_2_ gradient range across the microchannels, according to simulation results of the computational model.

Our experimental data indicate that this device is suitable to generate a stable oxygen gradient, and that the gradient affects cell phenotype and gene expression in a similar fashion to what occurs in the hepatic lobule, with differential expression of drug-metabolizing enzymes (CYP3A4). A major effect on glycogen metabolism is observed in the different hepatocytes derived from hESCs, according to their relative position in the oxygen gradient. Glycogen storage changes according to the level of oxygen, recapitulating the specialization of the hepatocytes in an *in vivo* microenvironment. Moreover, transcripts that are differentially expressed across the liver sinusoid, such as GLUL, CPS1, CCND1, CCNA and ALB, are differentially expressed in the monolayer of cells across the microfluidic channels, according to their specific position/O_2_ concentration. We could not assess a functional differential localization for CTNNB protein (beta-catenin) across the microfluidic oxygen gradient, due to limitation in retrieving a sufficient quantity of cells from microchannels for proteomic analyses. Despite this, for the proliferation marker Ki67 we could show a direct correspondence between the mRNA (Fig. 4) and the protein level (Suppl. Fig. 3A).

## Conclusions

Together, our results show how an oxygen gradient alone can significantly promote the establishment of a functional hepatic metabolic zonation during hESCs differentiation towards hepatocytes. These findings highlight oxygen as a relevant driving signal for the establishment of the zonation between the periportal and perivenous region in the hepatic sinusoid.

Despite this consideration, we cannot exclude that other factors can be involved in the induction of liver zonation in our system. It is known that, *in vivo*, dynamic gradients of different modulators such as cytokines and/or signaling molecules contribute to the zonation effect^1^.

In this perspective, it is possible that cells undergoing zonation can produce and release in the extracellular environment factors promoting zonation in our system. The production of factor(s) that can influence in an autocrine fashion the neighboring cells has not been investigated here. However, our model has the potential to allow investigation also in this sense.

In conclusion, the device developed can physiologically induce the differentiation of hepatocytes which, in the future, may be used to deepen our knowledge about the physiology/pathology of periportal and perivenous hepatocytes and possibly to reconstitute the diseased liver tissue.

## Materials and Methods

### Microfluidic device design and fabrication

Microfluidic device (referred as ‘chip’) designed to run experiments in triplicate, is composed of culture chambers and gas channels. Each culture chamber (18 × 1.5 × 0.25 mm) is separated for the entire length by a 100 µm membrane from a gas channel on both the sides. The gas channel (250 µm wide and 250 µm high) has one entrance that divides in two parallel channels in order to distribute the gas mixture equally for the three culture channels. The chip has been fabricated with standard photo-lithography techniques^27^ using SU8 2100 (Microchem). The chip has been replicated using PDMS (Sylgard 184, Dow Corning) in the rate 10:1 base:cure agent. Holes in the channels have been created using a 21G dispensing needle (Small Part Inc). The chip has been bonded via plasma treatment (Harrick Plasma, 120 s) on a soda lime glass slide (Menzel Glaser). A PDMS medium reservoir of about 200 µL has also been plasma-bonded to the designated outlet side. We also covered the upper surface of the chip with 100 µm coverslip glass slide in order to reduce water permeation and increase gradient efficiency.

### Computational model

For computer-assisted microfluidic chip design, we implemented a computational model to simulate the effect of gas stream concentration into the channel during steady state. The mathematical model was implemented and solved using Comsol Multiphysics (version 4.4 CLK) by diffusion with flux, insulation and concentration boundary condition. We simulated a chip section composed of culture chamber and gas mixture chambers supposing to place the chip in normoxic environment modifying the boundary condition in terms of concentration. Since PDMS has high gas permeable diffusion, a stable oxygen concentration can be generated by oxygen diffusion through a membrane that separates gas and flow chambers. We also studied the effect of insulation of the top of the chip on the concentration gradient distribution. For the simulation, we assumed that at higher gas flow rate (>0.1 mL/min) oxygen concentration in the center and in the wall of the gas channel was constantly maintained for the entire length of the channels and equal to the inlet concentration. The oxygen diffusion parameters [m^2^/s] used in the simulation are: in PDMS 4.10^−09^, in PBS 2.10^−09^ and in Air 2.00^−05^.

### Generation and characterization of the oxygen gradient

Every microfluidic channel is enclosed between a pre-humidified gas stream without oxygen (O_2_ 0%, N_2_ 95%, CO_2_ 5%) simulating the hypoxic condition, and the atmospheric oxygen in the incubator (O_2_ 21%), which acts as an infinite atmospheric oxygen reservoir. The hypoxic mixture is generated via mass flow meters (Bronkhorst) controlled through software FlowDDE32. The gas mixture constant flow is 1 mL/min. Before entering in the gas channels, the mixture is humidified and warmed by gurgling through a tank filled with distilled water placed at 37 °C. All the fluidic and gas connections were made with PTFE tubes (Cole Parmer). The gradient generation and stability were validated using a 100-mM solution of tris(4,7-diphenyl-1,10-phenanthroline)ruthenium(II) dichloride complex (Ru(ddp) – Sigma-Aldrich). Fluorescence of Ru(ddp) was detected by an inverted fluorescent microscope Leica DMI600B equipped with a mercury short-arc reflector lamp (Leica) excited at BP 450–490 nm and acquisition at LP 590. The Ru(ddp) solution was loaded inside the culture channels using a syringe pump (Harvard Apparatus) using 3-ml syringes (BD Plastic) at a flow rate of 500 nL/min. Images were taken every 5 sec at constant flow rate under steady state conditions to avoid Ru(ddp) bleaching. To correlate the fluorescence intensity, we applied the Stern-Volmer equation^28^:

$$\frac{{I}_{0}}{I}=1+{k}_{q}{\tau }_{0}[{O}_{2}]$$

where ${I}_{0}$ and $I$ are the fluorescence intensities of Ru(ddp) probe in the absence and presence of oxygen, ${k}_{q}$ is the quencher rate coefficient (in m^3^/mol/s, derived from the Stokes-Einstein relation), ${\tau }_{0}$ is the unquenched excited-state lifetime (previously reported to be ~$6\cdot {10}^{-6}$ s^29^, and $[{O}_{2}]$ is the concentration of oxygen in solution in mol/m^3^, obtained from Henry’s law with a Henry’s constant in water at 37 °C of 5.1848 ∙ 10^9^ Pa^30^.

### Cell culture and differentiation protocol

The human embryonic stem cells used for the experiments are HES2 line obtained by the National Stem Cell Bank (Madison, WI) and MShef-3 obtained by UCL (London).

HES2 were cultured on gelatin-coated multiwells with mitomycin C-treated mouse embryonic fibroblasts (Chemicon) co-culture, in expansion medium DMEM F-12 supplemented with 20% KO serum, 10% MEF conditioned medium, 20 ng/mL basic fibroblast growth factor, 0.1 mM β-mercaptoethanol, 1% non-essential amino acid (NEAA) and 1% penicillin-streptomycin (Thermo Fisher). HES2 were passaged to new feeder with trypsin 0.25% (Thermo Fisher). For MEFs depletion and cell differentiation, cells were passaged on matrigel reduced factor (BD Bioscience) coated channels of microfluidic device. MShef-3 cells were expanded feeder-free in mTeSR™1 Medium (Stemcell Tech) defined medium, on 0.5% matrigel coated plates. Cell suspensions were injected into channels and incubated overnight at 37 °C and 5% CO_2_ atmosphere without perfusion, to allow cell adhesion to substrate.

Cell differentiation started when cells reached a confluence level of approximately 70% by replacing stem cell expansion medium with endoderm induction medium for 3 days, containing RPMI 1640, 1X B27 supplement (both Thermo Fisher), 100 ng/mL human activin A (Peprotech), 50 ng/mL human wnt-3a (R&D Systems) and 1% penicillin-streptomycin. Medium was changed to differentiation medium, KO-DMEM with 20% KO serum, 1% NEAA, 1 mM glutamine, 0.1 mM β-mercaptoethanol, 1% penicillin-streptomycin (all Thermo Fisher) and 1% DMSO (Sigma-Aldrich) for 5 days. At this stage of differentiation (day 8), oxygen gradient is applied to culture system. Cells were subsequently cultured for other 9 (or more) days in maturation medium containing L15 medium (Sigma-Aldrich), 8.3% FBS (Thermo Fisher), 8.3% tryptose phosphate broth, 10 µM hydrocortisone-21-hemisuccinate, 1 µM insulin (all Sigma-Aldrich), and 2 mM glutamine with 10 ng/ml human hepatocyte growth factor and 20 ng/ml human oncostatin-M (both R&D Systems). The medium in the microchannel was changed every 12 h during differentiation^18^.

### RNA extraction and qRT-PCR

For cell extraction, PDMS upper parts of the microfluidic chips were peeled off from the glass slides with adherent cells. Channels monolayers were manually divided and picked with a glass pipette on the stereomicroscope to separate the cells growing in the low O_2_ side of the channel from the high O_2_ side.

Total RNA was then extracted from the collected hepatocyte-like cells by using 0.5 ml of Trizol Reagent (Thermo Fisher) per sample or pooled samples. Chloroform (Sigma-Aldrich) 0.1 mL was added to Trizol for 3 min and centrifuged at 12500 g for 15 min at 4 °C. Aqueous supernatant was collected and diluted 1:1 with 70% ethanol. Total RNA was extracted from solution using RNeasy Mini Kit (Qiagen), following the manufacturer’s instructions. The Nanodrop ND 1000 (Cell Euroclone) is used to determinate RNA concentration and quality. 500 ng of total RNA was reverse transcribed using MMLV reverse transcriptase (Thermo Fisher). The following primers are used for quantitative real time PCR (qRT-PCR):

β-Catenin, FW 5′-GCT TTC AGT TGA GCT5 GAC CA-3′, REV 5′-CAA GTC CAA GAT CAG CAG TCT-3;

Cyclin D1, FW 5′-CCG TCC ATG CGG AAG ATC-3′; REV 5′-CCT CCT CCT CGC ACT TCT GT-3′;

Cyclin A, FW 5′-GTC AGA GAG GGG ATG GCA T-3′, REV 5′-CCA GTC CAC CAG AAT CGT G-3′;

Albumin, FW 5′-CCT GTT GCC AAA GCT CGA TG-3′, REV 5′-GAA ATC TCT GGC TCA GGC GA-3′;

CPS1, FW 5′-GGC CAT CCA TCC TCT GTT GC-3′, REV 5′-GCT AAG TCC CAG TTC ATC CA-3′;

GLUL, FW 5′-GCC ATG GGG GAG GAG AAT-3′, REV 5′-ACT GGT GCC GCT TGC TTA GT-3′;

MKI67, FW 5′-GCC TGC TCG ACC CTA CAG A-3′, REV 5’– GCT TGT CAA CTG CGG TTG C-3′;

28S, FW 5′-TGG GAA TGC AGC CCA AAG-3′, REV 5′-CCT TAC GGT ACT TGT TGA CTA TGC-3′.

For the qRT-PCR the amplification steps were the following: pre-denaturation at 95 °C for 10 min, 40 cycles of amplification with denaturation at 95 °C for 15 s, annealing at proper temperature for 60 s and extension at 72 °C for 30 s. An extension step at 72 °C for 10 min and a dissociation stage (95/60/95 °C for 15 s each) is finally added. The levels of target mRNAs have been normalized to the levels of the 28S rRNA.

### Immunofluorescence assay

Immunofluorescence on endoderm and hepatocyte-like cells was performed after fixing cells with 4% PFA for 15 min. Definitive endoderm was marked with antibody SOX-17 1:100 (AF1924, R&D) incubated in 5% horse serum (Thermo Fisher), PBS/Triton X-100 0.3% for 1 h at room temperature (RT). Hepatic endoderm was marked with α-fetoprotein 1:250 (A8452, Sigma-Aldrich). Immature hepatocytes were stained with HNF-4α 1:50 (sc-8987, Santa Cruz Biotechnology). Mature hepatocyte-like cells were stained with albumin 1:100 (MAB1455, R&D), and CYP3A4 (GTX60577, Genetex) incubated in 10% FBS, PBS/Triton X-100 0.1% overnight at 4 °C. For secondary antibody and staining we used Alexa Fluor 594 or 488, phalloidin-488, Hoechst (all Thermo Fisher) for 1 h at 37 °C.

Cell viability assay (Live/Dead kit, Thermo Fisher) was performed on microfluidic differentiated cells. Cells were washed with basal DMEM-F12, incubated with 3 μM ethidium homodimer-1 (stains dead cells in red), 3 μM calcein AM (stains live cells in green) and 4 μM Hoechst (stains cell nuclei in blue) for 45 min at 37 °C, washed with DMEM-F12 and analyzed at fluorescence microscope.

### Fluorescence quantification

Channel width was virtually divided in 3 sections (upper, middle, lower), and fluorescent pictures were acquired at the Leica DMI6000 B microscope (see Suppl. Fig. 1). From each photo, two regions of interest (ROI) were considered, for a total of 6 ROI for each microchannel section. ROI fluorescence was quantified using LasAf Lite software image analyzer, and relative fluorescent unit (R.F.U.) were plotted against ROI position across channel width.

### Periodic acid schiff (PAS) stain and quantitative assay

To assess glycogen storage in hepatocyte-like cells, we performed the Periodic Acid Schiff (PAS) staining using Periodic Acid Schiff kit (Sigma-Aldrich) according to manufacturer’s instructions. Briefly, hepatocyte-like cells were fixed in 4% PFA in PBS 1X for 15 min at RT, washed with PBS 1X (negative controls were treated with diastase 1 mg/mL for 15 min) and stained with periodic acid for 5 min at RT. After that, cells were washed with distilled water, stained with Schiff’s reagent for 15 min at RT and then washed again with distilled water. Finally, cells were thoroughly washed with distilled water prior to microscopic examination.

To quantify the glycogen storage in terms of PAS+ area, microscopic images were first converted from RGB to HSV color space, and then only the saturation component was used for further processing. Saturation was thresholded according to a parameter that distinguishes between PAS+ and PAS− pixels. This parameter was manually chosen by comparing the saturation levels of PAS and diastase-treated PAS pictures and kept constant in the analysis of all the images.

### Statistical analysis

Data are presented as mean ± SE or ± SD. T-test was held out by Instat 2.0 and Graphpad 6.1 software to determine the statistical differences between the analyzed groups. The degree of significance has been set at (p < 0.05).

## Supplementary information

Supplementary Figures

Supplementary Video 1

Supplementary Video 2

## Data Availability

The datasets generated during and/or analyzed during the current study are available from the corresponding author on reasonable request.

## References

[1] Kang YBA, Eo J, Mert S, Yarmush ML, Usta OB (2018). Metabolic Patterning on a Chip: Towards *in vitro* Liver Zonation of Primary Rat and Human Hepatocytes. Sci Rep.

[2] Jungermann K, Katz N (1989). Functional specialization of different hepatocyte populations. Physiol Rev.

[3] Godoy P (2013). Recent advances in 2D and 3D *in vitro* systems using primary hepatocytes, alternative hepatocyte sources and non-parenchymal liver cells and their use in investigating mechanisms of hepatotoxicity, cell signaling and ADME. Arch Toxicol.

[4] Gebhardt R, Matz-Soja M (2014). Liver zonation: Novel aspects of its regulation and its impact on homeostasis. World J Gastroenterol.

[5] Burke ZD (2018). Spatiotemporal regulation of liver development by the Wnt/beta-catenin pathway. Sci Rep.

[6] Gebhardt R (1992). Metabolic zonation of the liver: regulation and implications for liver function. Pharmacol Ther.

[7] Bralet MP, Branchereau S, Brechot C, Ferry N (1994). Cell lineage study in the liver using retroviral mediated gene transfer. Evidence against the streaming of hepatocytes in normal liver. Am J Pathol.

[8] Kietzmann T, Dimova EY, Flugel D, Scharf JG (2006). Oxygen: modulator of physiological and pathophysiological processes in the liver. Z Gastroenterol.

[9] Du Y (2009). Rapid generation of spatially and temporally controllable long-range concentration gradients in a microfluidic device. Lab Chip.

[10] Adler M, Polinkovsky M, Gutierrez E, Groisman A (2010). Generation of oxygen gradients with arbitrary shapes in a microfluidic device. Lab Chip.

[11] Song JW (2009). Microfluidic endothelium for studying the intravascular adhesion of metastatic breast cancer cells. PLoS One.

[12] Huh D (2007). Acoustically detectable cellular-level lung injury induced by fluid mechanical stresses in microfluidic airway systems. Proc Natl Acad Sci USA.

[13] Allen JW, Bhatia SN (2003). Formation of steady-state oxygen gradients *in vitro*: application to liver zonation. Biotechnol Bioeng.

[14] Allen JW, Khetani SR, Bhatia SN (2005). *In vitro* zonation and toxicity in a hepatocyte bioreactor. Toxicol Sci.

[15] Sato A, Kadokura K, Uchida H, Tsukada K (2014). An *in vitro* hepatic zonation model with a continuous oxygen gradient in a microdevice. Biochem Biophys Res Commun.

[16] Lee-Montiel FT (2017). Control of oxygen tension recapitulates zone-specific functions in human liver microphysiology systems. Exp Biol Med (Maywood).

[17] Whitesides GM, Ostuni E, Takayama S, Jiang X, Ingber DE (2001). Soft lithography in biology and biochemistry. Annu Rev Biomed Eng.

[18] Giobbe GG (2015). Functional differentiation of human pluripotent stem cells on a chip. Nat Methods.

[19] Luni C (2016). High-efficiency cellular reprogramming with microfluidics. Nat Methods.

[20] Tachikawa M (2018). Liver Zonation Index of Drug Transporter and Metabolizing Enzyme Protein Expressions in Mouse Liver Acinus. Drug Metab Dispos.

[21] Torre C, Perret C, Colnot S (2011). Transcription dynamics in a physiological process: beta-catenin signaling directs liver metabolic zonation. Int J Biochem Cell Biol.

[22] Jungermann K, Kietzmann T (1996). Zonation of parenchymal and nonparenchymal metabolism in liver. Annu Rev Nutr.

[23] Hay DC (2008). Highly efficient differentiation of hESCs to functional hepatic endoderm requires ActivinA and Wnt3a signaling. Proc Natl Acad Sci USA.

[24] Hannan NR, Segeritz CP, Touboul T, Vallier L (2013). Production of hepatocyte-like cells from human pluripotent stem cells. Nat Protoc.

[25] Asplund A (2016). One Standardized Differentiation Procedure Robustly Generates Homogenous Hepatocyte Cultures Displaying Metabolic Diversity from a Large Panel of Human Pluripotent Stem Cells. Stem Cell Rev.

[26] Martinez I (2008). The influence of oxygen tension on the structure and function of isolated liver sinusoidal endothelial cells. Comp Hepatol.

[27] Luni C, Michielin F, Barzon L, Calabro V, Elvassore N (2013). Stochastic model-assisted development of efficient low-dose viral transduction in microfluidics. Biophys J.

[28] Stern OaV (1919). O. The extinction period of fluorescence. Phys. Z.

[29] Wang XD, Wolfbeis OS (2014). Optical methods for sensing and imaging oxygen: materials, spectroscopies and applications. Chem Soc Rev.

[30] Luni C (2011). Design of a stirred multiwell bioreactor for expansion of CD34+ umbilical cord blood cells in hypoxic conditions. Biotechnol Prog.

