# Supplementary material for: In vitro metabolic zonation through oxygen gradient on a chip
Source: Sci Rep. 2019 Sep 19;9:13557. doi: 10.1038/s41598-019-49412-6 (PMC6753109; doi:10.1038/s41598-019-49412-6)
Supplement: Supplementary file 1 — Supplementary Figures [file 41598_2019_49412_MOESM1_ESM.pdf]

In vitro metabolic zonation through oxygen gradient on a chip

Federica Tonon [1,¶], Giovanni Giuseppe Giobbe[2,3¶], Alessandro Zambon[3], Camilla Luni[4], Onelia Gagliano[3,6], Annarosa Floreani[5], Gabriele Grassi[1] and Nicola Elvassore[2,3,4,6*]

[1] Dept. of Life Sciences, University of Trieste, Trieste, 34127, Italy

[2] Stem Cell and Regenerative Medicine Section, UCL GOS Institute of Child Health, London, WC1N 1EH, UK

[3] Dept. of Industrial Engineering, University of Padova, Padova, 35131, Italy

[4] Shanghai Institute for Advanced Immunochemical Studies (SIAIS) ShanghaiTech University, Shanghai, 201210, China

[5] Dept. of Surgery, Oncology and Gastroenterology, University of Padova, Padova, 35121, Italy

[6] Venetian Institute of Molecular Medicine, Padova, 35129, Italy

* Corresponding author nicola.elvassore@unipd.it

¶ First co-authorship: Federica Tonon and Giovanni Giuseppe Giobbe contributed equally to this work.

Supplementary Figures

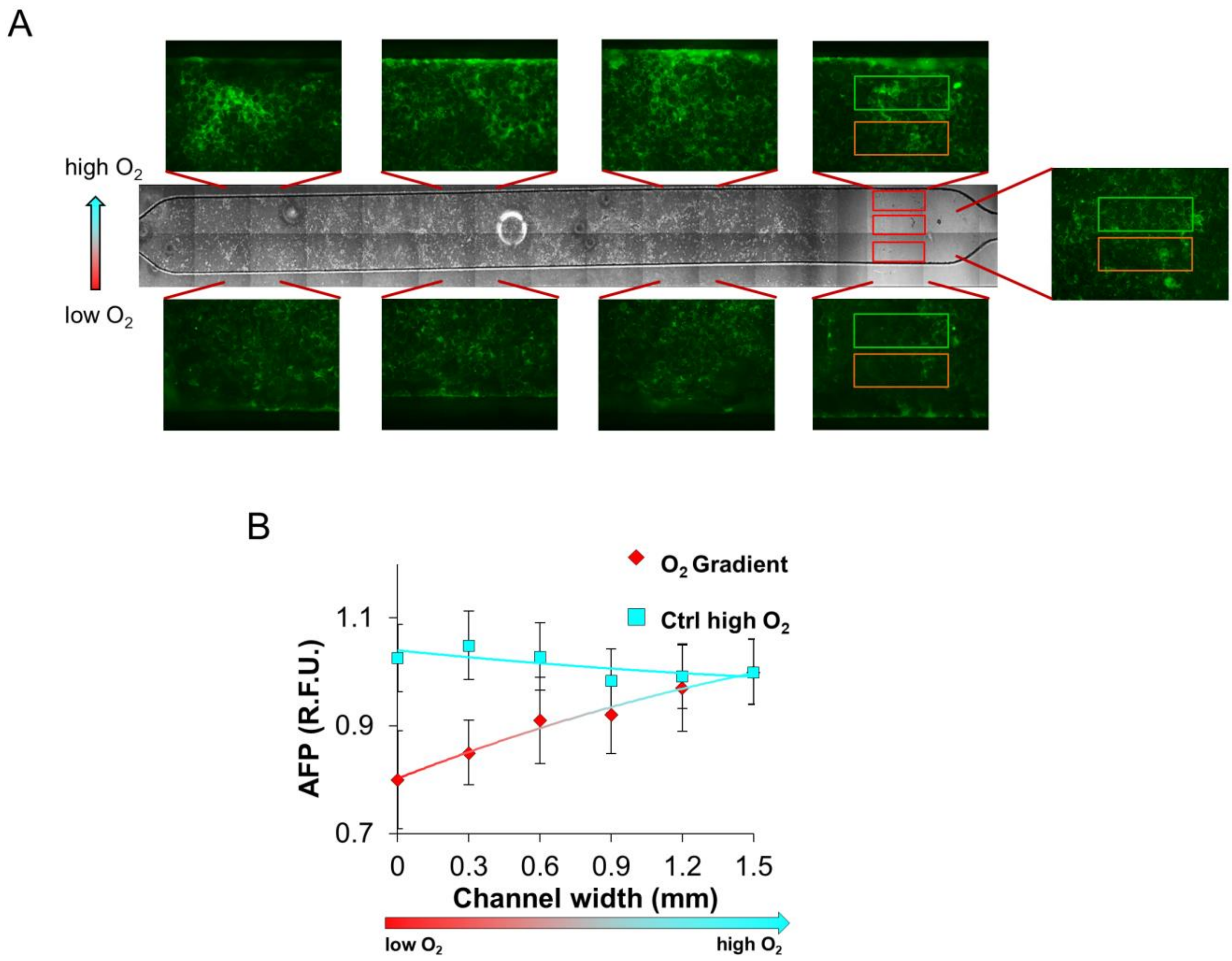

Supplementary Figure 1. (A) Alpha-fetoprotein immunostaining (green) at the different sides of the microfluidic channel with oxygen gradient applied from day 1 (hESCs) to day 8 (hepatic endoderm) of differentiation, and the 6 regions of interest (ROI) selected for fluorescence quantification. (B) Relative fluorescence unit plot of control chip not exposed to gradient and cultured at standard high O_2 (21%) and gradient chips that shows differential expression of alpha-fetoprotein across the gradient.

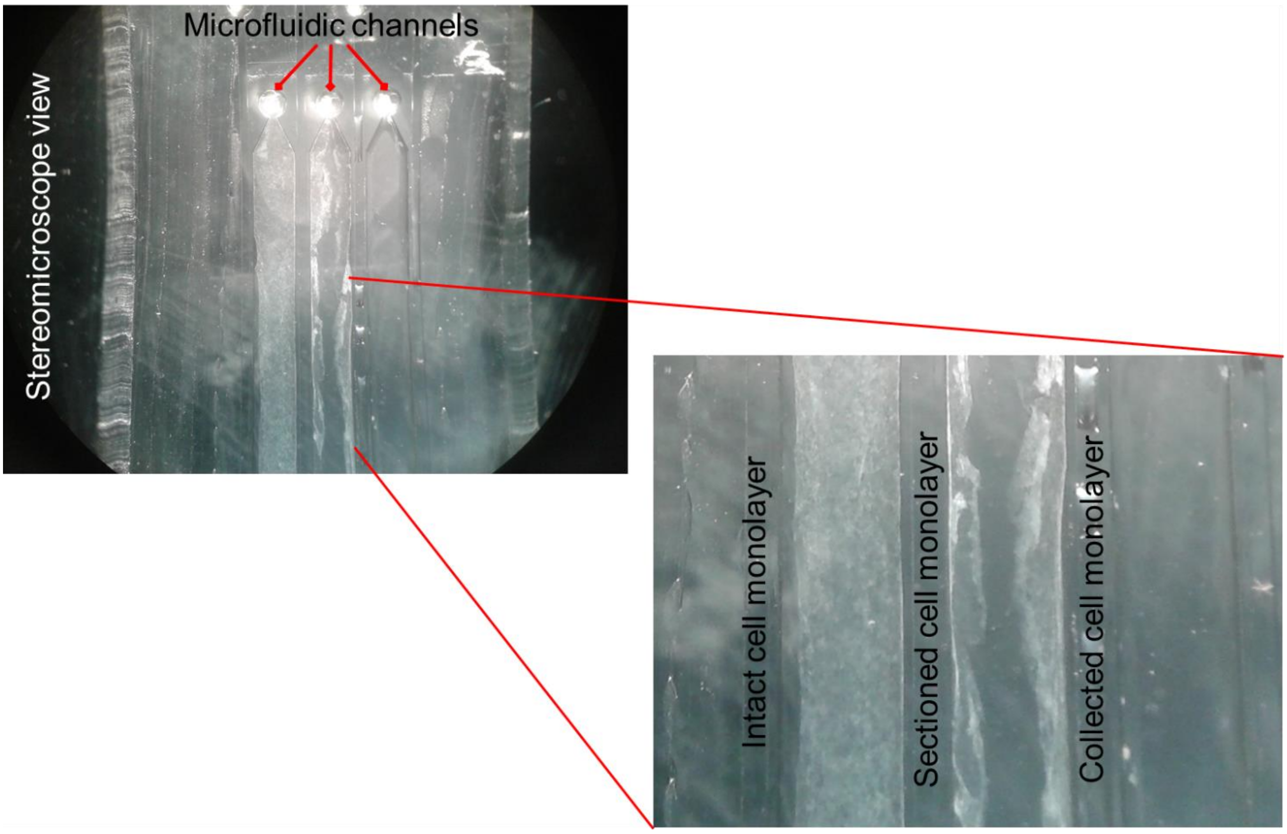

Supplementary Figure 2. Stereomicroscope manual picking of the two sides of the hESC-hepatocyte monolayer within the microfluidic channel for RNA collection.

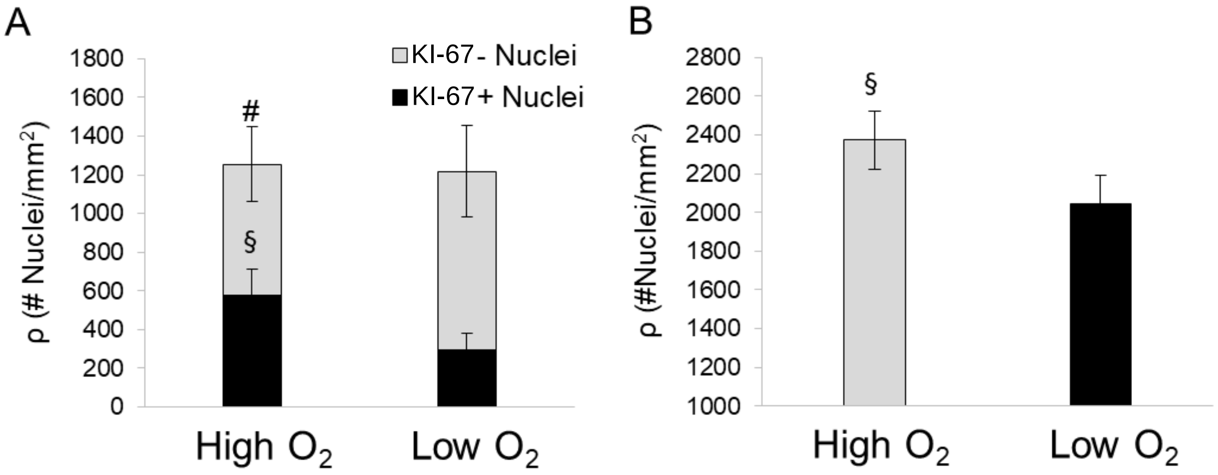

Supplementary Figure 3. Evaluation of the proliferative potential of hepatocyte-like cells differentiated and grown on plate or on a microfluidic device. (A) Quantification of KI-67 immunostaining in hepatocyte-like cells obtained differentiating hESCs grown on a plate at low (<5%) or high (21%) oxygen. Cells differentiated at high O_2 show a higher amount of KI-67 positive nuclei compared to cells grown at low oxygen. Data are shown as mean ± SD, n=25. p§ < 0.0001 respect to KI-67+ nuclei at low (<5%) oxygen and p# < 0.05 respect to KI-67 – nuclei at low (<5%) oxygen. (B) Nuclei counting of hepatocyte-like cells obtained from hESC grown on a microfluidic device. On the side with higher O_2, more nuclei were observed compared to the other side of the chamber (low O_2). Data are shown as mean ± SD. n=55. p§ < 0.0001 respect to low O_2.
